# Supplementary material for: Quantitative Analysis of Signal Heterogeneity in the Hepatobiliary Phase of Pretreatment Gadoxetic Acid-Enhanced MRI as a Prognostic Imaging Biomarker in Transarterial Chemoembolization for Intermediate-Stage Hepatocellular Carcinoma
Source: Cancers (Basel). 2023 Feb 15;15(4):1238. doi: 10.3390/cancers15041238 (PMC9954181; doi:10.3390/cancers15041238)

Table S1. Univariate analysis of risk factors associated with OS up to 2 years after treatment among patients with intermediate-stage HCC.

	Univariate analysis	
	P-value	HR (95% CI)
Age ≥ 80 years	0.2635	2.351 (0.5254–10.52)
Etiology of liver disease	0.9954	1.006 (0.1315–7.696)
Coefficient of variation	0.1543	1973 (0.05767–67530000)
AFP ≥ 200 ng/mL	0.1156	2.543 (0.7952–8.131)
Up-to-7 out	0.005301	5.227 (1.634–16.71)
Up-to-11 out	0.05858	3.445 (0.9561–12.41)
Child–Pugh score 7	0.1567	2.96 (0.6592–13.3)
ALBI grade 2	0.1045	3.456 (0.7733–15.45)
PT <70%	0.02848	5.423 (1.195–24.61)
Totalbilirubin ≥2.0 mg/dL	0.9978	0.0000001091 (0.000–Inf)
Albumin <3.5 g/dL	0.07879	2.667 (0.8933–7.964)
Post-TACE MTA	0.5238	0.6144 (0.1375–2.746)
SEX	0.2585	2.371 (0.5304–10.59)

Figure S1. Kaplan–Meier analysis of OS in the group scanned by 1.5T between two groups divided based on each explanatory variable.

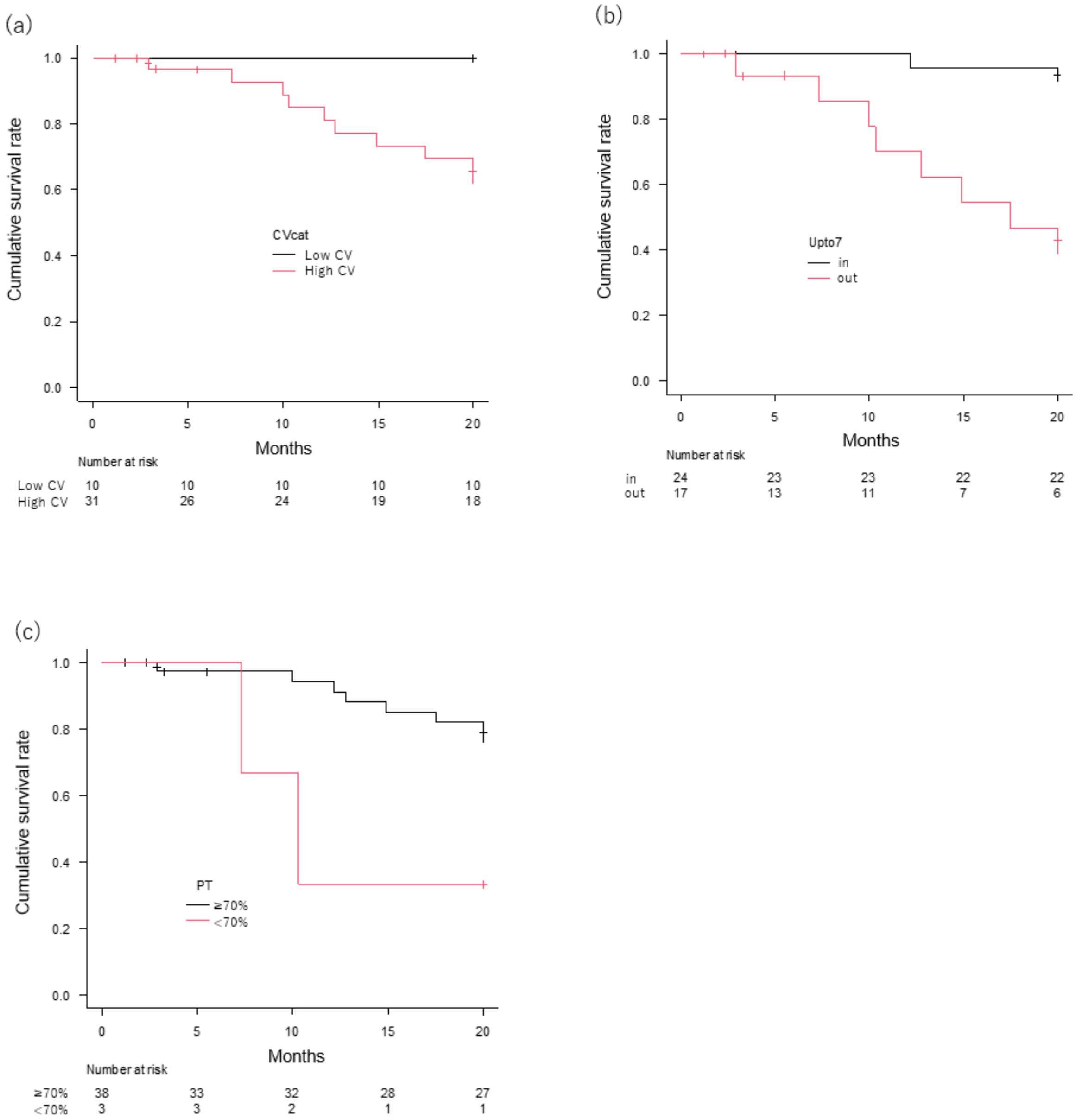

Figure S2. Kaplan–Meier analysis of OS in the group scanned by 3.0T between two groups divided based on each explanatory variable.

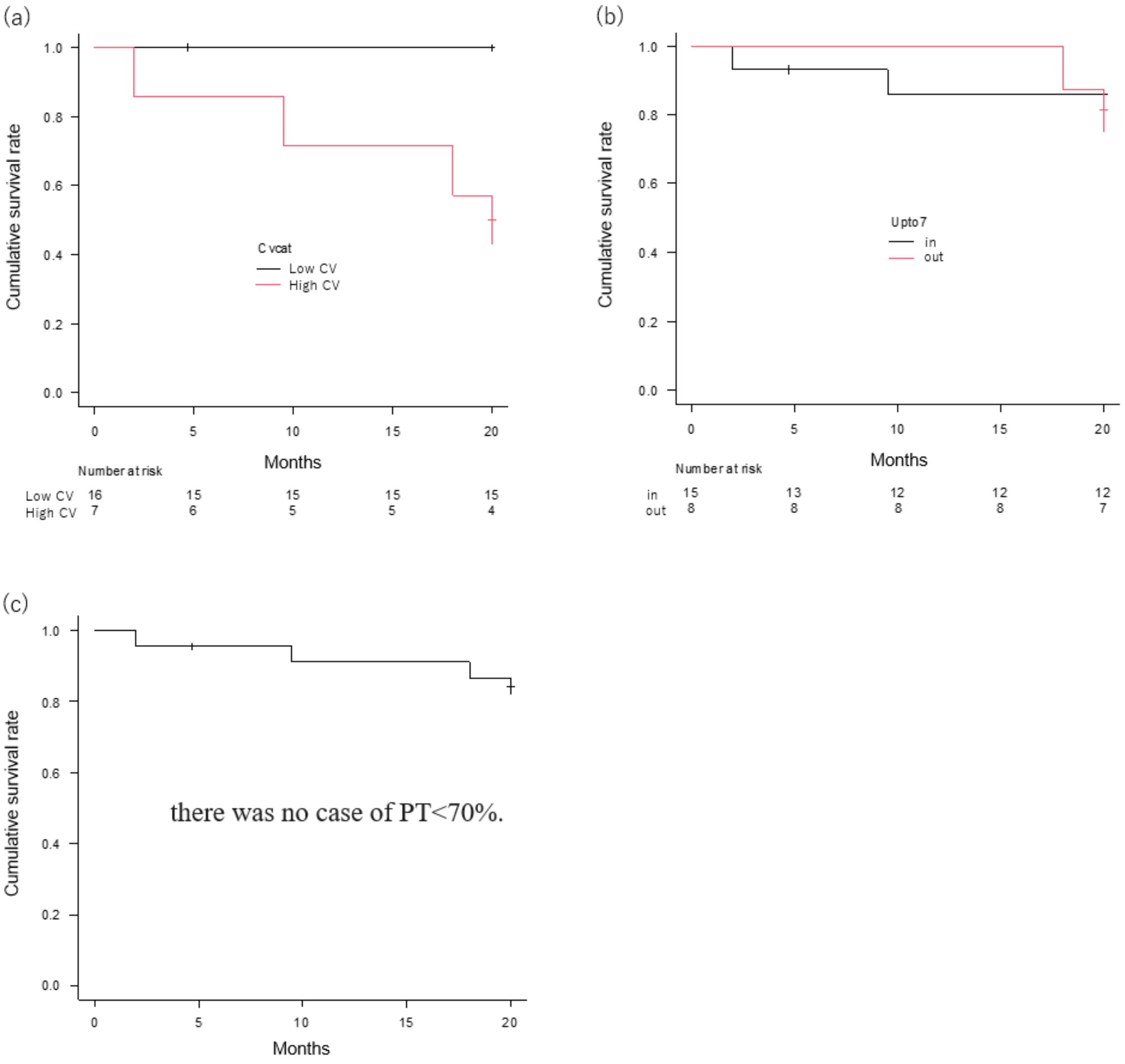

Supplement: Supplementary file 1 [file cancers-15-01238-s001.zip › cancers-2135537-supplementary.pdf]
